# DNA Nanostructure as an Efficient Drug Delivery Platform for Immunotherapy

**DOI:** 10.3389/fphar.2019.01585

**Published:** 2020-01-28

**Authors:** Qingjia Chi, Zichang Yang, Kang Xu, Chunli Wang, Huaping Liang

**Affiliations:** ^1^ State Key Laboratory of Trauma, Burns and Combined Injury, Research Institute of Surgery, Daping Hospital, Army Medical University, Chongqing, China; ^2^ Hubei Key Laboratory of Theory and Application of Advanced Materials Mechanics, Department of Mechanics and Engineering Structure, Wuhan University of Technology, Wuhan, China; ^3^ Department of Cardiovascular Surgery, Union Hospital, Tongji Medical College, Huazhong University of Science and Technology, Wuhan, China; ^4^ “111” Project Laboratory of Biomechanics and Tissue Repair, Bioengineering College, Chongqing University, Chongqing, China

**Keywords:** DNA nanostructure, immunotherapy, drug delivery, DNA cage, DNA hydrogel

## Abstract

Immunotherapy has received increasing attention due to its low potential side effects and high specificity. For instance, cancer immunotherapy has achieved great success. CpG is a well-known and commonly used immunotherapeutic and vaccine adjuvant, but it has the disadvantage of being unstable and low in efficacy and needs to be transported through an effective nanocarrier. With perfect structural programmability, permeability, and biocompatibility, DNA nanostructures are one of the most promising candidates to deliver immune components to realize immunotherapy. However, the instability and low capability of the payload of ordinary DNA assemblies limit the relevant applications. Consequently, DNA nanostructure with a firm structure, high drug payloads is highly desirable. In the paper, the latest progress of biostable, high-payload DNA nanoassemblies of various structures, including cage-like DNA nanostructure, DNA particles, DNA polypods, and DNA hydrogel, are reviewed. Cage-like DNA structures hold drug molecules firmly inside the structure and leave a large space within the cavity. These DNA nanostructures use their unique structure to carry abundant CpG, and their biocompatibility and size advantages to enter immune cells to achieve immunotherapy for various diseases. Part of the DNA nanostructures can also achieve more effective treatment in conjunction with other functional components such as aPD1, RNA, TLR ligands.

## Introduction

Immunotherapy means a method of treating diseases by managing the native immune system of the body. As a relatively novel therapeutic strategy, immunotherapy has received increasing attention due to its low potential side effects and high specificity (Martin-Liberal et al., 2017). For example, cancer immunotherapy has made great progress in recent decades, especially for the therapies of recurrent and metastatic cancer (Sharma and Allison, 2015; Young, 2017; Li et al., 2019). There are a variety of immunotherapeutic strategies for different diseases, such as vaccine-based therapies (Wu, 2012; Banchereau and Palucka, 2017) and CpG-based therapies (Friedberg et al., 2005; Melief and van der Burg, 2008; Mohri et al., 2012).

Although clinical results are encouraging, immunotherapy is effective only for a small portion of the disease (Naran et al., 2018; Pauken et al., 2019). This is partly due to the higher requirements of immunotherapy for drug carriers, such as precise targeting, biocompatibility, and controlled release. In recent years, the researchers have put a lot of effort to develop nanotechnology-based methods to improve immunotherapy for various diseases (Look et al., 2010; Look et al., 2014; Shukla and Steinmetz, 2016; Liu et al., 2018; Al-Halifa et al., 2019). Nanomaterials-based therapeutics with unique properties may help address some of the key technical challenges in immunotherapy. Nanomaterials have been widely used to transport a variety of biologically active immune-related antigens and adjuvants (Zhu et al., 2014). The small size of nanomaterials promotes penetration into mesenchyme and mucosal barrier surrounding the antigen-presenting cells (APCs), resulting in efficient cellular uptake. Besides, some well-designed nanocarriers can serve as a transport platform for a variety of therapeutic cargoes simultaneously (Zhu et al., 2014).

DNA nanostructures bind to these therapeutic molecules in immunotherapy. Due to its high degree of structural programmability, permeability, and biocompatibility, DNA nanostructures are among the most promising candidates for delivery of immune pharmaceuticals (Jin et al., 2017; Yang et al., 2019). In our previous studies, we paid attention to the biophysical aspect of the structure as a kind of biomacromolecule (Chi and Jiang, 2012; Chi et al., 2013), the molecular mechanism of immunomodulation and immunotherapy of small molecule drugs (Tian et al., 2015; Chai et al., 2016; Zhu J. et al., 2018). DNA-based nanotechnology has become a new way to create biocompatible, well-defined scaffolds because of their biological origin, unparalleled structural precision, and customizability, allowing a wide range of self-assembled structures to be built in a bottom-up manner (Meng et al., 2016; Hu et al., 2018; Zhang et al., 2018). With its high degree of programmability, it is convenient to build complex DNA nanostructures with precisely defined geometries and shapes. Complementary base pairing provides excellent programmability for DNA, making it ideal for building complex nanostructures (Kumar et al., 2016). Due to the natural programmability of materials, different types of nanostructures have been established, including DNA cages, DNA particles, DNA polypods, and DNA hydrogel. All of these features open up new opportunities to advance the development of DNA-based nanodiagnostics (Sau et al., 2018; Tyagi and Subramony, 2018).

## DNA Nanostructure for Immunotherapy

Immunostimulatory and immunomodulatory nucleic acids are common adjuvants in the immunotherapy of various diseases (Sau et al., 2018; Tyagi and Subramony, 2018). For example, CpG and poly I:C are capable of reacting with different TLR-like receptors to elicit a strong systemic immune reaction, and they can also be used as vaccine components for immunotherapy (Yu et al., 2018). These immunomodulatory nucleic acids have been applied to treat psoriasis, lupus and arthritis, thrombosis (Yu et al., 2018). Oligodeoxynucleotides (ODN) containing an unmethylated CpG motif are considered to be effective immunotherapeutic vaccine adjuvants to help achieve effective therapeutic applications because it can stimulate Toll-like receptors 9 (TLR9). CpG has been studied in clinical trial groups for melanoma immunotherapy, metastatic breast cancer, and glioblastoma multiforme. The stimulation of TLR9 stimulates immune-relevant cells like dendritic cells (DCs), macrophages, and B cells to produce pro-inflammatory cytokines. The nanostructure of DNA is commonly used as a delivery platform for CpG, such as DNA tetrahedron (Li et al., 2011) and tubular DNA origami (Li et al., 2011). After being taken up by cells and recognized by TLR9, these pro-inflammatory cytokines are secreted to achieve immunotherapeutic effects of various diseases. Binding of TLR9 triggers an NF-κB-related signaling cascade to promote the expression of pro-inflammatory cytokines, namely tumor necrosis factor-ɑ (TNF-ɑ), interleukin-6 (IL-6), interleukin- 12 (IL-12) (Huang et al., 2017), co-stimulatory factors like CD80 and CD86. These events promote survival and proliferation of APCs and promote Th1 immunostimulatory response while inhibits Th2 adaptive immune responses.

DNA nanostructure-based vaccines are promising vectors for immunizing various human diseases, including hepatitis B (Huang et al., 2017), tuberculosis (Tang et al., 2015), Alzheimer disease (Matsumoto et al., 2013), and malaria parasites (Tyagi et al., 2012). The DNA-based immunization is successful in initiating cellular and humoral immune responses without triggering immunity against the vector (King et al., 2015). There exist other advantages for DNA-based vaccines. They can polarize T cells and trigger a Th1 immune response (Lysén et al., 2019). Compared to protein-based vaccines, DNA vaccines are more stable and show a longer shelf life, making them both advantageous in terms of preparation, storage, and transportation (Stenler et al., 2014; Hobernik and Bros, 2018).

However, the instability and low payload of common DNA assemblies limit the related applications. Therefore, DNA nanostructures with a robust structure, high drug payload, and good cellular uptake are highly desirable. Recent reviews have focused on the overall description of DNA nanostructures in biomedical applications, while the content of immunotherapy is briefly mentioned (Stenler et al., 2014; Hobernik and Bros, 2018). However, few people discuss the role of DNA nanostructures in immunotherapy in detail. The paper will focus on reviewing DNA nanostructures that have stable structures, high payloads, and good immunotherapeutic effects on various diseases.

## Wireframe DNA Cages

**Figure 1:**
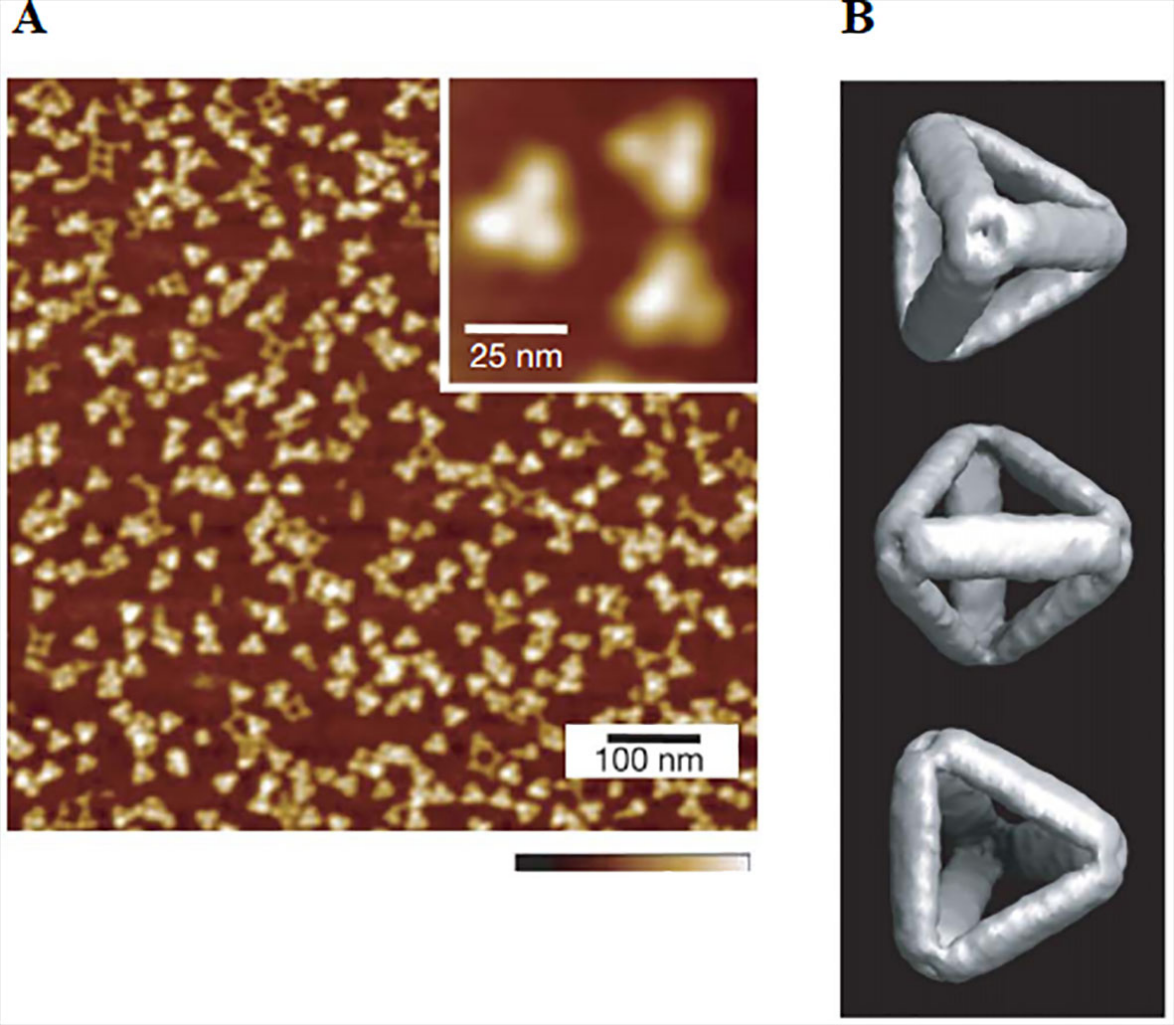
Observation and characterization of tetrahedral DNA by **(A)** AFM and **(B)** CyroEM [reproduced with permission from (He et al., 2008)].

DNA cages refer to wireframe architectures assembly from DNA strands(Chen and Seeman, 1991; Wang et al., 2019). Various DNA cages include DNA polyhedrons (Wang et al., 2019) and DNA nanotube (Jorgenson et al., 2017; Mohammed et al., 2017) were reported. DNA polyhedrons represent a 3D cage-like compact structure which is stable and easily absorbed by cells. Due to structural closure, they are compact, mechanically strong, size-tunable and noncytotoxic (Jorgenson et al., 2017; Mohammed et al., 2017). Among them, DNA tetrahedron is the most commonly seen one (He et al., 2008; Liang et al., 2014), as demonstrated in **Figure 1**. Now they have been applied to load various immune moieties including CpG (He et al., 2008; Liang et al., 2014), peptides (Xia et al., 2016), for applications in different therapies. Great efforts have been made in the targeted modification to promote efficiency and prevent side effects.

**Figure 2:**
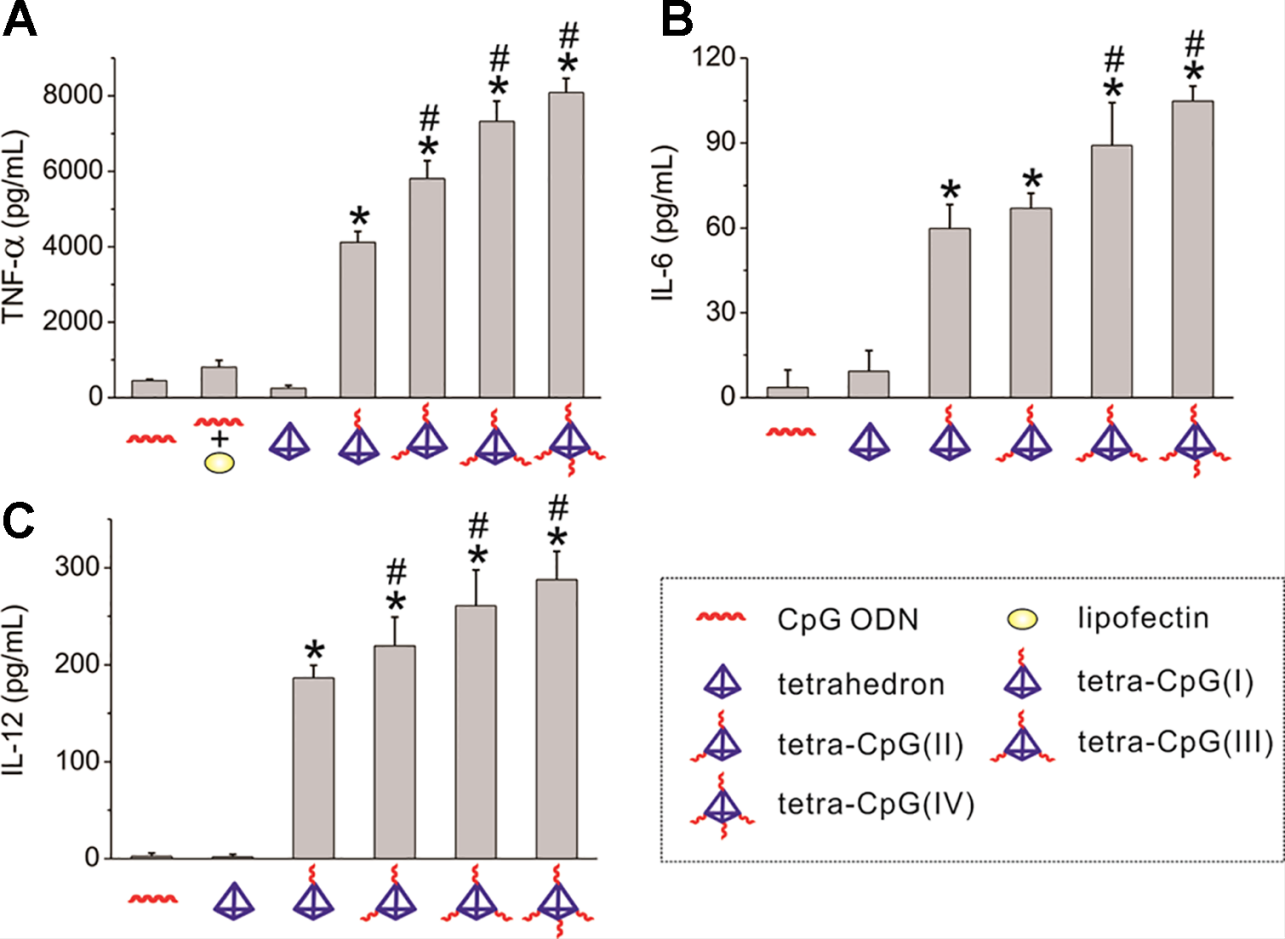
Cytokine release from RAW264.7 cells stimulated by DNA tetrahedron. Secretion of **(A)** TNF-ɑ, **(B)** IL-6, and **(C)** IL-12 under the action of CpG ODNs and DNA tetrahedron [reproduced with permission from (Li et al., 2011)]. TNF-α, tumor necrosis factor-α; ODNs, Oligodeoxynucleotides. *P < 0.001 significantly different from CpG ODN and tetrahedron. ^#^P < 0.05 significantly different from tetra-CpG(I).

Wireframe nanostructures, such as DNA tetrahedra, constructed from DNA-lipid micellar nanoparticles compared to Watson-Crick base pairings are capable of assembling more CpG ligands, and in the equivalent case require fewer nucleotides. The immune effects of tetrahedral CpG molecules are most significant compared to other structures. Ohtsuki et al. designed three different structures, including CpG tetrahedron, tetrapodna, and tetragon. They found that CpG tetrahedrons enter cells most efficiently, and induce the largest amount of TNF-ɑ compared to the latter two (Ohtsuki et al., 2015). Consequently, DNA tetrahedra are often used to transport CpG to bring about immune response efficiently. Authors use DNA tetrahedron as a nanocarrier for targeted delivery of CpG (Li et al., 2011). The results show that DNA tetrahedral nanostructures can remain intact in the serum and living macrophage-like cells for a duration at least several hours. The tetrahedron carrying several CpG motifs enters the cells without a transfection agent to trigger a strong immune response, as shown in **Figure 2**. The results demonstrated that the mammalian immune system can accept DNA tetrahedral safely as a delivery system.

DNA tetrahedral nanostructures can mimic the complex structure of VLPs, providing an ideal multifunctional platform for building DNA vaccines. The proximity of the antigen and adjuvant is rather beneficial to enhance the vaccine immunogenicity. It has been shown the direct attachment of CpG ODNs to antigen-induced strong immune responses (Klinman et al., 1999). In vivo observations have shown that CpG-containing oligonucleotides can increase the production of serum antibody by 10 times. However, the direct connection has limitations in the construction of more complex vaccines. Liu et al. first used DNA tetrahedrons as scaffolds to assemble synthetic vaccine complexes containing a model antigen, streptavidin (STV) and CpG (Liu et al., 2012). The vaccine complex is similar to natural virus particles in the geometry. Compared to the control group of naked STV and the directly linked ODN-STV, DNA tetrahedrons can promote the combinational delivery of CpG and antigens to cancer cells, constituting an important prerequisite for immune response. As a result, a strong and long-lasting immune response was triggered *in vivo* without rejection of the nanocarrier. The co-assembly system of antigen-adjuvant was safe because anti-dsDNA antibodies against tetrahedral structures did not appear in mouse serum for a dozen of days after secondary immunization.

**Figure 3:**
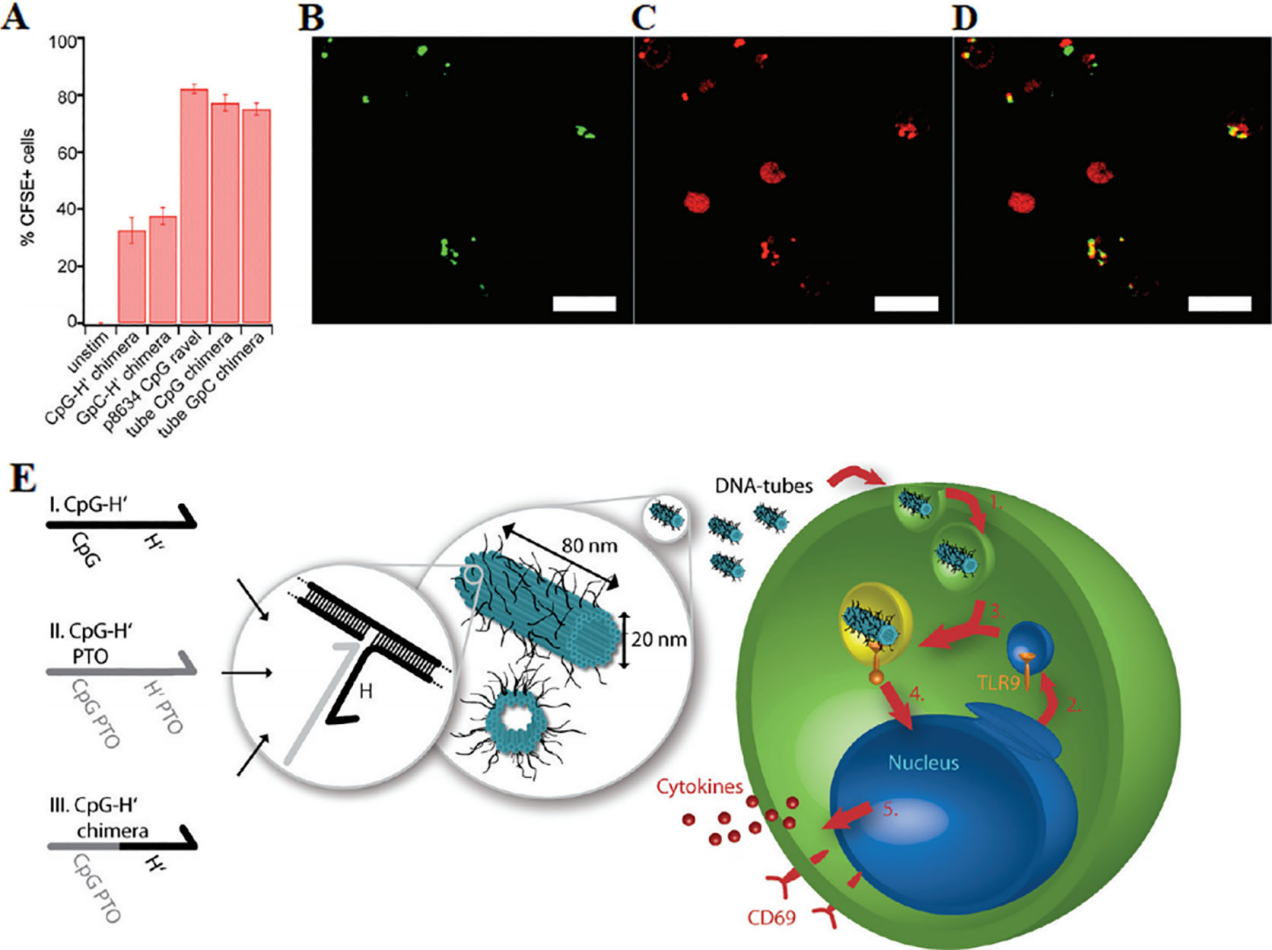
Uptake of CpG-decorated DNA nanostructures by macrophages. **(A)** A comparison of absorption of CpG bound by different DNA nanostructures **(B)** Green indicates DNA origami tubes chimera III with FITC. **(C)** Red indicates lysosomes. **(D)** Merge of A and B. Scale bars: 10 μm. **(E)** A depiction of 30-helix DNA origami nanotube incorporated by 3 different kinds of CpG-H's with (I) unmodified phosphate backbone, (II) phosphorothioate (PTO)-modified backbone, and (III) partly PTO-modified backbone. Blue cylinders refer to double helices; black lines refer to possible binding sites for CpG ODNs [reproduced with permission from (Schüller et al., 2011)]. ODNs, Oligodeoxynucleotides.

DNA nanotubes constructed from DNA origami can also be used to build a biocompatible delivery platform of CpG. The DNA origami technology allows a long DNA single strand that is folded into a specific geometry by about several hundred oligonucleotides. The method constructs the DNA assembly to exhibit a highly complex shape with nanometer-scale precise component alignment on its surface (Linko and Dietz, 2013). The DNA origami structure maintains its structural integrity when exposed to a variety of endonucleases. It has been reported a 8634-bp single-stranded DNA (ssDNA) scaffold containing hundreds of short fibers was folded into a hollow DNA nanotube in which 62 binding sites of CpG ODNs are presented (Schüller et al., 2011), as shown in **Figure 3E**. The structural characteristics of DNA nanotube result in up to 62 drug binding sites. DNA nanotubes can provide much more drug targets than ordinary DNA nanostructures. The CpG-bearing DNA nanotube has better immune stimulation to spleen cells and lower cytotoxicity than liposome-based delivery, as demonstrated in **Figures 3A–D**. The Liedl group demonstrated that microinjection of CpG-decorated DNA nanotubes in the skeletal muscle of mice is effective in eliciting immunogenic responses (Sellner et al., 2015). The DNA nanotube was internalized and located in the endosomes of the tissue-resident macrophages within a few minutes. Microinjection of CpG modified DNA nanotube instead of ordinary DNA nanotube or CpG ODNs significantly recruits macrophages into muscle tissue and activate the inflammatory pathway in cells. These findings indicated that DNA nanotubes serve as an impressive transport platform for targeting and activating macrophages.

## DNA-Based Nanoparticles

### Spherical Nucleic Acids

Spherical nucleic acids (SNAs) has two components, including a dense radially surrounding nucleic acid shell and a solid or hollow nanoparticle core. Compared with linear nucleic acid, SNA has many advantages. First of all, the affinity of SNA to complementary nucleic acids is higher than that of linear counterpart due to its special geometry, thereby increasing the stability of the structure (Seferos et al., 2009). Second, SNA can enter a variety of cells and with excellent cellular uptake in the absence of an auxiliary transfection agent (Williams, 2013). Finally, SNAs is composed of biologically compatible materials and are not toxic to cells (Melamed et al., 2018), making SNA a powerful tool in numerous biomedical applications. The nucleic acid shell of SNA can serve as a high-affinity binder for different classes of ligands to fulfill particular purposes, making SNA a powerful platform for the application of molecular diagnostic and (Halo et al., 2014), gene regulation (Zheng et al., 2012) and immunomodulatory therapy (Banga et al., 2017a).

**Figure 4:**
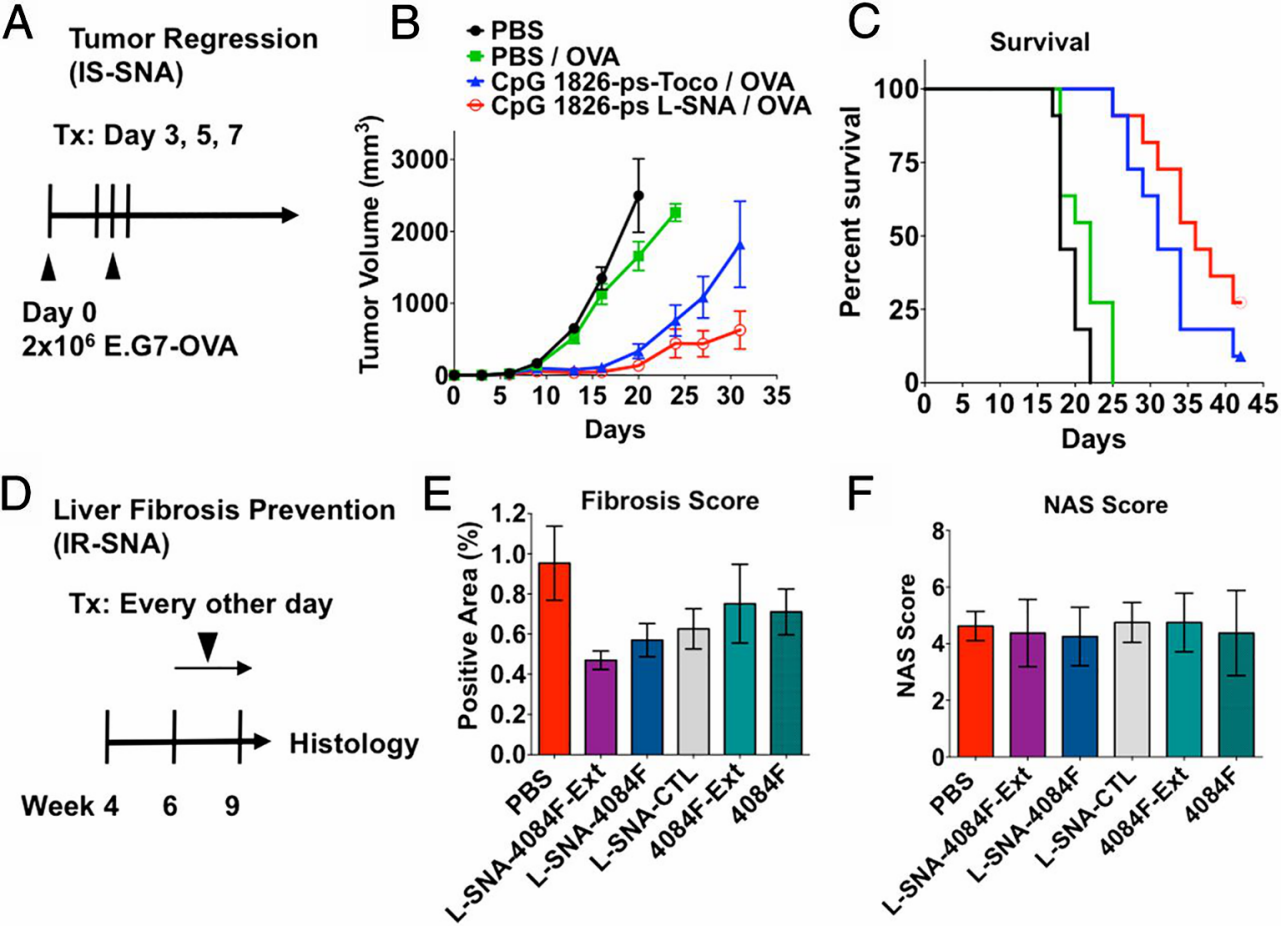
Therapeutic effect of SNA on tumor and liver fibrosis *in vivo* **(A)** SNA strongly suppresses tumor **(B)** Changes of tumor volume with time under the action of LSNA and other control groups and **(C)** Curve of survival percentage. **(D)** immunoregulatory SNAs show enhanced treatment of liver fibrosis in mice with NASH, as measured by **(E)** fibrosis score and **(F)** nonalcoholic fatty liver disease activity (NAS) score [reproduced with permission from (Radovic-Moreno et al., 2015)]. SNA, spherical nucleic acids; LSNA, Liposomal spherical nucleic acid.

The 3D structure of SNA, rather than the nanoparticle core, is the key to its versatility (Banga et al., 2017a). The radial alignment of nucleic acid and the 3D structure of the SNA with increases the surface area, bringing about abundant drug binding sites. Great effort is put on designing new SNA with biocompatible organic nanoparticles cores, including liposomes and polymer micelles and other biodegradable materials (Zhang C. et al., 2015; Banga et al., 2017a; Sprangers et al., 2017), which increase additional immune functionality and therapeutic effect. The immunomodulatory function of SNA is particularly notable. SNA has been regarded as an immunomodulator that binds TLR 7, 8, and 9 to their sequence-identified nucleic shells (Zhang C. et al., 2015; Banga et al., 2017a; Sprangers et al., 2017). Due to its unique 3D structure, CpG attached to SNA increases its affinity for its target to promote immune regulation. Investigators have developed immunostimulatory CpG-coated SNA with a core of gold nanoparticles. These SNA-induced mouse macrophages and human peripheral blood mononuclear cells produce higher proinflammatory cytokines than their soluble counterparts (Radovic-Moreno et al., 2015). The immunomodulatory SNA reduced fibrosis in a mice model of nonalcoholic steatohepatitis (NASH) by 40%–51% (**Figures 4D–F**). Immunostimulatory SNAs increased the production of IL-12 and interferon-γ (IFN-γ) by 10 times in mice, confirming that these DNA-based structures have the systemic immunostimulatory capacity to regulate the immune system. Furthermore, a strong immune response was elicited in the mouse when the antigen was loaded onto the immunomodulatory SNA, finally resulting in enhanced anti-tumor efficacy. **Figures 4A–C** shown that SNA carrying the antigen resulted in significant and sustained remission of tumor growth in mice and doubled survival rate.

The main disadvantages of gold particles based SNA include difficulty in degradation and high cost, limiting their applications further. Compared with gold particles based SNA, Liposomal spherical nucleic acids (LSNAs) LSNA has the advantage of biocompatibility and at the same time has the general properties of the latter (Banga et al., 2014), and is therefore often used in immunotherapy. LSNA is more potent than linear nucleic acid in activating immune cells like macrophages and DCs (Guan et al., 2018). Liposomal forms have entered the Phase 1b/2 human clinical trial. Radovic-Moreno et al. also designed liposome-based immunostimulation of LSNA carrying CpG and comparing them to CpG-carrying liposomes (Radovic-Moreno et al., 2015). LSNA was about three times more potent than liposome CpG as indicated from the activated B cells.

**Figure 5:**
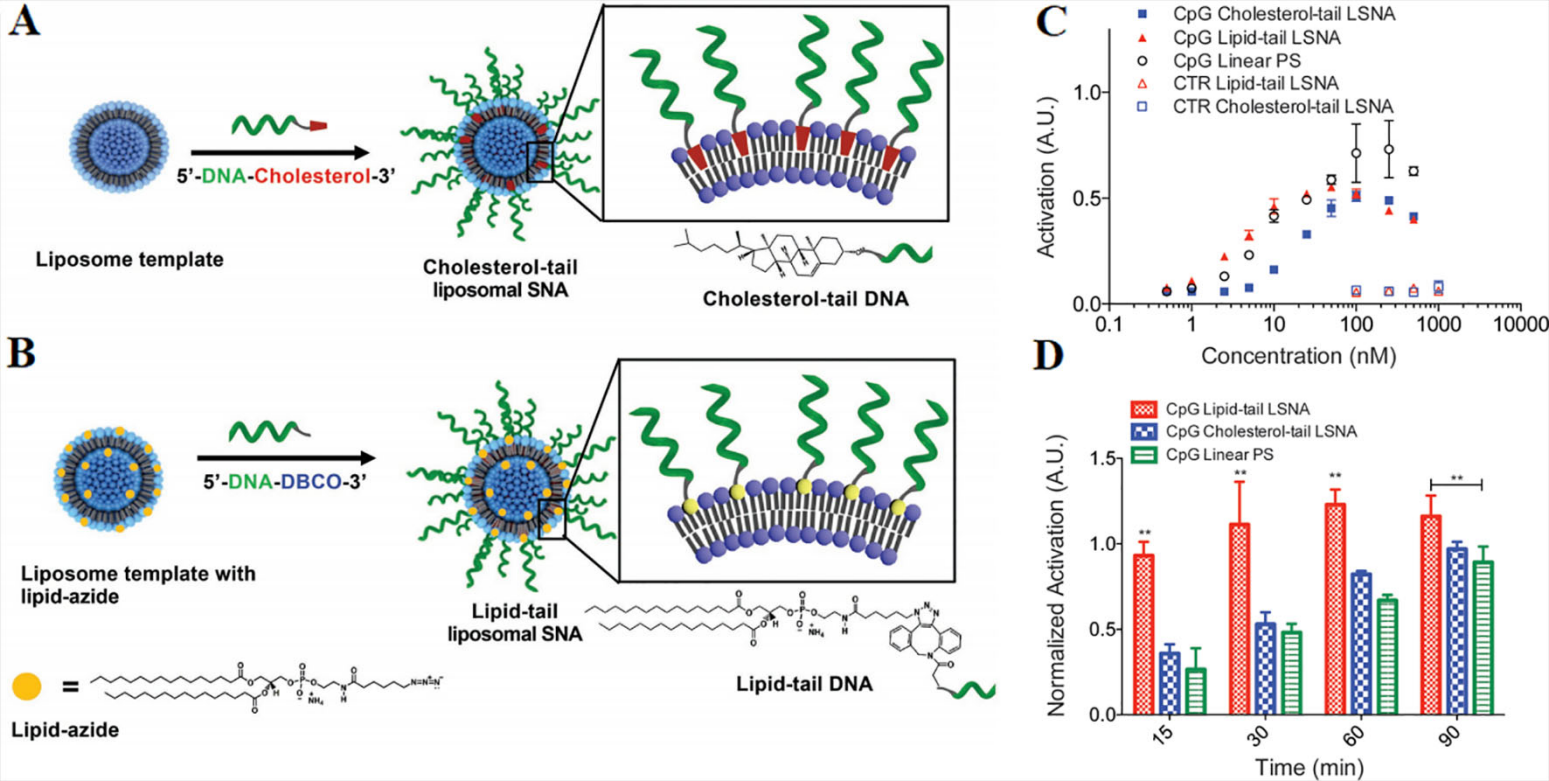
A comparison of two different preparation methods of LSNA **(A)** cholesterol-tail DNA **(B)** DNA lipid-tail. Stimulation of macrophages by CpG-incorporated LSNAs as characterized by change of **(C)** concentration **(D)** time. (*P < 0.01) [reproduced with permission from (Meckes et al., 2017)]. LSNA, Liposomal spherical nucleic acid. **P < 0.01 in comparision with CpG linear PS.

The synthesis method of LSNA is generally to anchor a nucleic acid modified with a hydrophobic component such as cholesterol to a lipid bilayer of a liposome template, as shown in **Figure 5A**. Nevertheless, the mobility of the liposome nucleus and the hydrophilic nucleic acid shell make the structure inherently less stable, limiting the widespread applications of LSNA (Reddy et al., 2012). The problem of stability become one of barrier for the use of LSNA. The increased stability of lipid-tail LSNA should keep the structure intact and be absorbed by the cells efficiently (Choi et al., 2013). Anchoring DNA with a lipid tail to the core of the liposome can improve the stability of traditional LSNA (**Figure 5B**) (Choi et al., 2013). LSNA synthesized with lipid-modified DNA results in a twofold increase of oligonucleotide loading, which should be equivalent to a larger immunotherapeutic payload. Moreover, the modification increased the stability of the structure and triggers faster cellular internalization and more intense immune activation. Compared with cholesterol-tail LSNA, macrophages also showed enhanced lipid-tail LSNA uptake (**Figures 5C, D**). Compared with cholesterol tail analogs, lipid tail LSNA showed moderately increased activity at lower concentrations and was able to activate macrophages more quickly, which may be the result of faster uptake by cells.

Activation of multiple receptors of cells is a common feature of many inflammation-mediated diseases, including sepsis, rheumatoid arthritis, liver fibrosis (Gao et al., 2017). Therefore, the ability of simultaneously targeting multiple TLRs on the cell membrane could enhance the treatment of these inflammation-mediated diseases (Gao et al., 2017). LSNA can achieve the goal successfully due to its versatile nucleic acid functionality. Liposomes co-encapsulating ligands of TLR-9 (CpG ODNs) and TLR-3 [poly(I:C)] enhanced cellular uptake and pro-inflammatory cytokine production and improved macrophage bactericidal activity (Bayyurt et al., 2017). Encapsulation of OVA antigen into liposome vesicles produced a durable anti-cancer immune response. And *in vivo* experiments showed that tumor progression was significantly inhibited in mice. Similar immunostimulatory activities are also present in human peripheral monocytes. Other researchers designed a novel dual-targeted LSNA from a single-layered liposome core that delivers a nucleic acid that specifically inhibits TLR9 and a small molecule that for TLR4 inhibition (Ferrer et al., 2019). The results showed that dual TLR targeting LSNA strongly inhibited TLR-9 and TLR-4, respectively in primary mouse macrophages. These LSNAs have a prominent ability to reduce inflammation, and they can down-regulate relevant pro-inflammatory molecules. Dual-targeted LSNA showed up to 10-fold and 1,000-fold increases in TLR inhibition, in comparison with linear and small molecule treatments.

The disadvantage of traditional SNA is that its core material is not biocompatible and is not easily degraded. The biocompatibility and safety of materials of SNA core have been improved continuously. The cross-linked micelle core of immunostimulatory SNA can be made from an FDA-approved thermosensitive block copolymer, which makes SNA technology closer to clinical applications (Banga et al., 2017b). The CpG possessing a lipid tail is inserted into the hydrophobic region of the micelle, followed by chemical crosslinking to form a stable structure. These SNAs are more potent in cells, respectively, compared to linear CpG. The novel SNA based on a core of poly (lactic-co-glycolic acid) (PLGA) nanoparticle also exhibits good absorbability and is free to enter macrophage cells to activate toll-like receptors nine in a dose-dependent manner (Zhu S. et al., 2018).

### Hybrid DNA-Based Nanoparticles

Small particles of inorganic nanoparticles, such as Ca^2+^, Mg^2+^, and Mn^2+^ phosphates have good biocompatibility and are easily absorbed by APCs. Consequently, they are suitable as carriers for immune responses or vaccines (Bhakta et al., 2014; Lin et al., 2017). Small-sized calcium phosphate (CaPi) nanoparticles can be used as promissing immunoregulatory agents (Hadjicharalambous et al., 2015; Tenkumo et al., 2018). And the researchers observed smaller particles conjugated with DNA triggered strong immune responses with high transfection efficiency (Singh et al., 2000). DNA-encapsulated small-size magnesium phosphate nanoparticles also have higher transfection efficiencies *in vitro* and *in vivo* (Bhakta et al., 2005).

**Figure 6:**
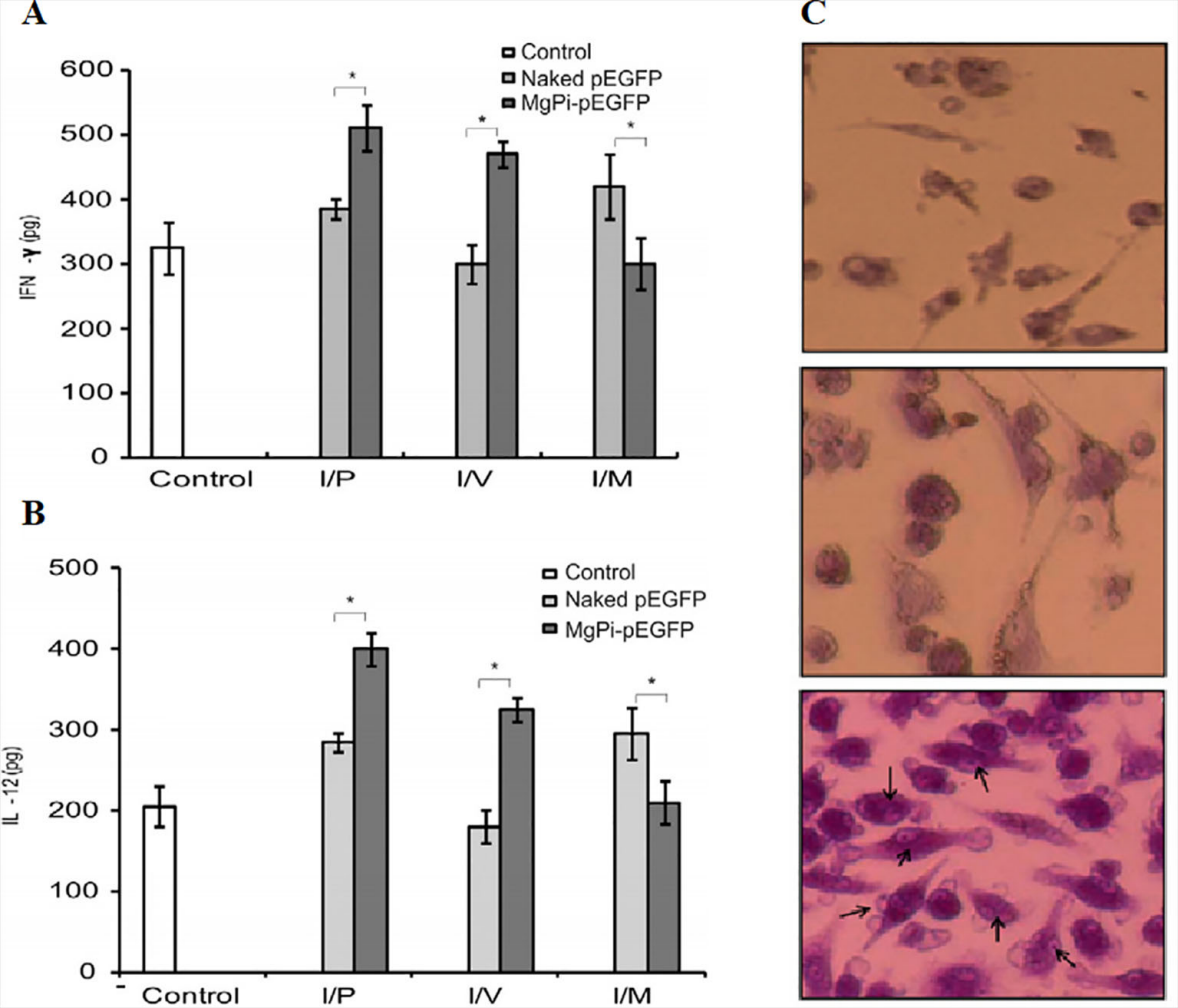
Production of **(A)** IFN-g and **(B)** IL-12 by stimulated splenocytes which are extracted from experimental animals (treated with control, pEGFP, and MgPipEGFP). *P < 0.05. **(C)** Activation of macrophage which was obtained from the spleen of 2 groups (naked and MgPi-encapsulated) The arrow refers to the phagocytosis of dead cells by splenocytes [reproduced with permission from (Bhakta et al., 2014)]. IFN-g, interferon-g; IL-12, interleukin-12; pEGFP, plasmid DNA expressing an enhanced green fluorescent protein.

**Figure 7:**
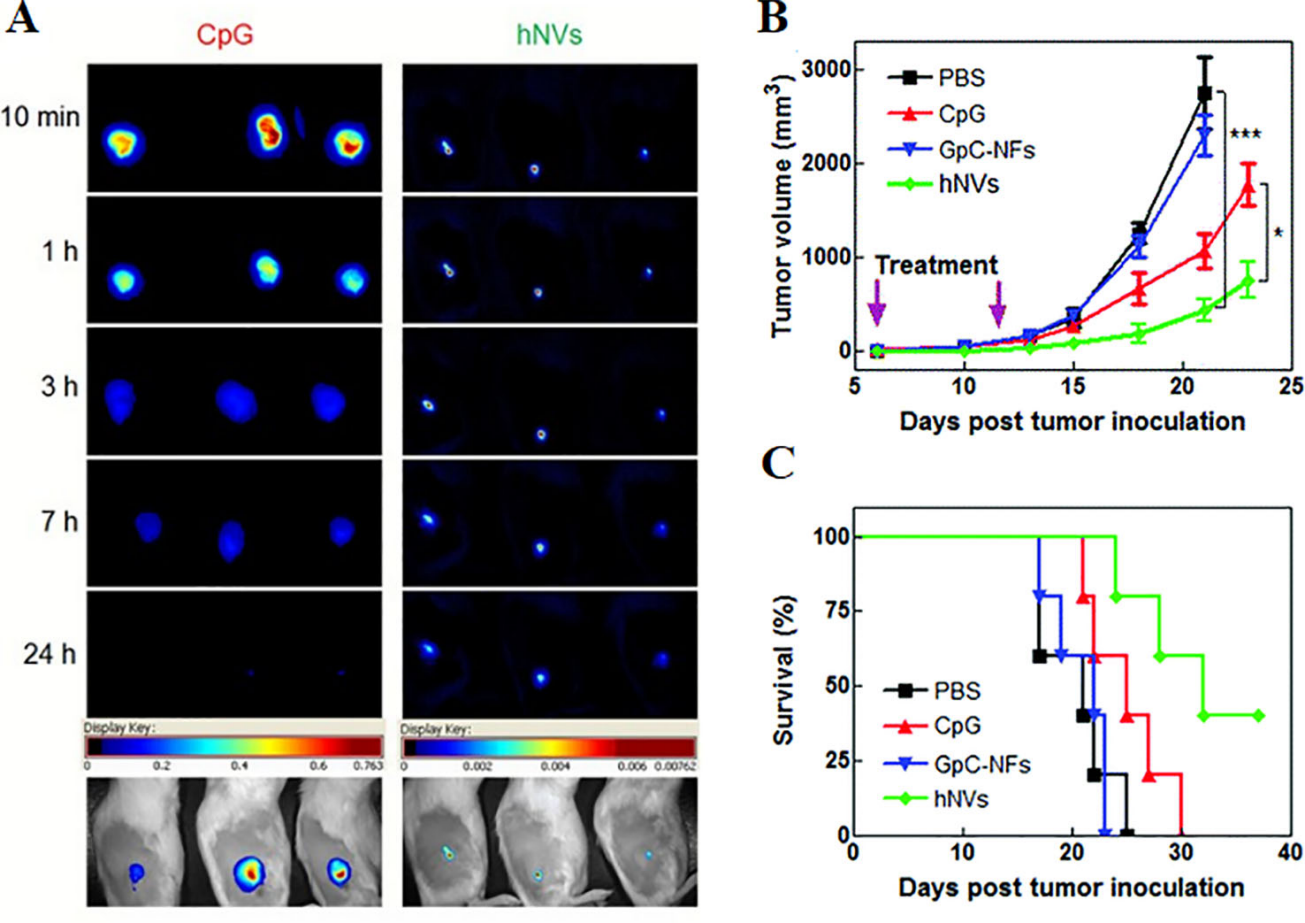
**(A)** Comparison of tumor retention time of hNVS and CpG by pharmacokinetics **(A)** The pharmacokinetics of hNVs or CpG were monitored by microscopical observation after subcutaneous injection. Shown at the bottom are representative overlayed images of mice and fluorescence. **(B)** Intratumoral injection of PBS, CpG molecules, control group GPC-NFS or hNVS. The changes in mouse tumor volume were compared to determine the immunotherapeutic effect of hNVs. (***p < 0.001, *p < 0.1). **(C)** Kaplan–Meier survival curve of mice of different groups [reproduced with permission from (Zhu et al., 2016)]. hNVS, hybrid nanovacciness.

DNA-encapsulated nanoparticles can constitute a safe and stable DNA vaccine formulation. The immunostimulatory efficacy of pegylated MgPi nanoparticles (MgPi-pEGFP) in a mouse model has been reported to be encapsulated with plasmid DNA expressing an enhanced green fluorescent protein (pEGFPa) (Bhakta et al., 2014). Compared to naked pEGFP, intravenously-administered MgPi-pEGFP nanoparticles induced enhanced IFN-γ and IL-12 expression (**Figures 6A, B**). A highly active macrophage response was also observed when the immunized mice were treated with the nanoparticles (**Figure 6C**). Zhu et al. introduced DNA-inorganic hybrid nanovaccines (hNVs) which were assembled from tandem CpG analogs as well as Mg_2_PPi (Zhu et al., 2016). hNVs show high load capacity and stability. In vivo pharmacokinetic observation revealed that hNVs demonstrated prolonged tumor retention in mice and reduced systemic toxicity as compared to that of tranditional CpG counterparts (**Figure 7A**). As a result, the hNVs can specifically inhibit tumor growth, and its anti-tumor efficacy is much stronger than other groups (**Figure 7B**). The survival rate of hNVs treatment was significantly higher than treatment with other regimens (**Figure 7C**). The experimental results *in vitro* also demonstrated that hNVs were efficiently internalized by DCs and macrophages, resulting in effective immune stimulation.

DNA nanoparticles containing metal materials also have degradation problems, limiting their biocompatibility. Taking organic material as the core of the nanoparticles, such as amphiphile structures, is an potential solution. Another method of constructing DNA nanoparticles utilizes hydrophobic units to form amphiphile-based micelle particles in an aqueous environment. When a hydrophobic unit such as a polymer or lipid is covalently linked to DNA, it undergoes microphase separation to self-assemble into a micellar structure (Kwak and Herrmann, 2011). These structures are not formed by Watson-Crick pairing, but by hydrophobic interactions, compared to the original DNA nano-objects. The soft material DNA nanoparticle has been used to deliver anticancer drugs (Alemdaroglu et al., 2008).

Although studies have shown that CpG can induce spleen DC activation [34,35], the role of CpG-conjugated DNA nano-objects in spleen DCs *in vivo* has not been well characterized. Jin et al. have successfully used DNA-lipid micelle nanoparticles for *in vivo* immune stimulation (Jin et al., 2017). The lipid-modified nucleotides and fluorescent probes are incorporated into the DNA strand to form uniform-sized DNA-lipid micelle particles, and the CpG-conjugated nanoparticles induce significant up-regulation of stimulator DC-derived stimulating molecules and cytokines. In vivo immunological results confirmed that systemic administration of DNA micelle particles effectively promoted up-regulation of costimulatory molecules and production of pro-inflammatory cytokines. Lipid-DNA nanoparticles that are further semi-filled with CpG fragments are capable of fully activating spleen DCs, while similar-sized DNA tetrahedra can only load fewer CpG chains with limited immunostimulation. DNA-lipid micelle nanoparticles can not only provide CpG adjuvant to spleen DC but can also load antigens. A specific DC that simultaneously acquires an adjuvant and antigens will induce a subsequent specific immune response against the corresponding pathogen.

**Figure 8:**
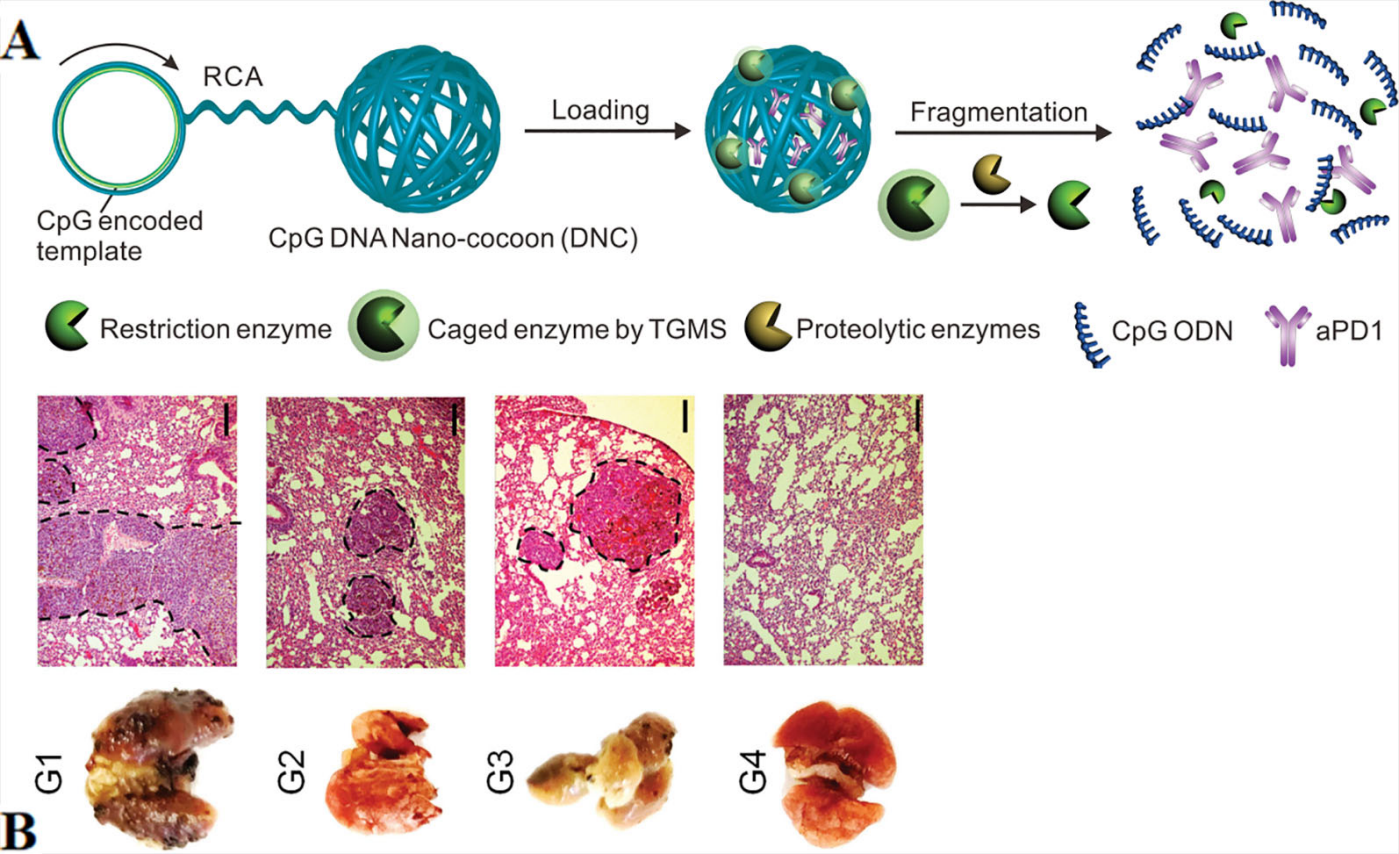
Simultaneous delivery of CpG ODNs and aPD1 by DNC in the inflammatory environment **(A)** DNCs containing aPD1 and restriction enzyme cleaves under the action of inflammatory environment, releasing CpG and aPD1. **(B)** In vivo antitumor effect of local injection of the delivery system of DNCs (Scale bar: 150 µm). [reproduced with permission from (Wang et al., 2016)]. ODNs, Oligodeoxynucleotides; DNCs, DNA nano-cocoons; aPD1, anti-PD-1 antibodies.

CpG can also achieve synergistic immunotherapy of cancers with other functional components such as neoantigens, aPD1, and RNA through nanoparticle-based carriers. Neoantigens are typically derived from tumor somatic mutations. They are selectively expressed in tumor cells to avoid autoimmunity against healthy tissues and cells.(Bobisse et al., 2016; Yi et al., 2018). Therefore, nanovaccines that co-deliver adjuvants and neoantigens have greater implications for tumor immunotherapy (Luo et al., 2017). The researchers reported self-assembled intertwining DNA-RNA nanocapsules (iDR-NC) for cancer immunotherapy. iDR-NC is a hybrid DNA-RNA nanostructure produced by combining rolling circle replication (RCR) and rolling circle transcription (RCT) in the reaction. The structure efficiently transfers CpG-encoded ssDNA, short hairpin RNA (shRNA), in conjunction with neoantigens to APCs synergistically (Zhu et al., 2017). The results showed that iDR-NC elicited the 8-fold release of peripheral CD8^+^ T cells than ordinary CpG counterpart and significantly inhibited colorectal tumor progression. An innovative DNA nano-cocoons (DNCs) have been reported to implement controlled release of CpG and anti-PD-1 antibodies (aPD1) under the stimulation of the inflammatory environment (Wang et al., 2016). **Figure 8A** shown DNCs assembled from ssDNA containing CpG sequences and cleavage sites of restriction enzyme, which is caged in amphiphilic nanoparticles and connected to DNC. The inflammatory microenvironment of the wound disassemble the cage and releases the enzyme, then digests DNC and finally releases the CpG fragment as well as aPD1. The continuous presence of CpG and aPD1 from DNC fragmentation synergistically promotes long-lasting T cell responses to treat melanoma (**Figure 8B**). Investigators further designed a PD-L1 trap to reduce the immune-related adverse effects of treatment of anti-PD-L1 monoclonal antibodies (Song et al., 2018). PD-L1 trap is built by loading plasmid DNA into nanoparticles of lipid-protamine-DNA (LPD). The combined presence of chemotherapeutics and the trap made a good anti-tumor effect with low side effects.

## Polypod-Like DNA Nanostructure

Besides dendrimers, Polypod-like nanostructure referred to another kind of branched nanoassembiles consist of structural body “trunk” together with many “legs”. Investigators have reported various types of polypod-like DNA nanostructure, including polypodna (Yata et al., 2015), DNA nanocentipede. Their structural advantages are their long backbone and numerous branch structures. Compared to ssDNA structures, the backbone structure of multi-legged DNA nanostructures provides structural stability, and the branched structure greatly increases drug binding targets. They potentially provide adequate binding sites due to the branched nature of the structure.

Polypod-shaped DNA is a DNA structure composed of three or more ODNs. Polypod-shaped DNA has several helix arms which are intersected at points, endowing abundant potential docking sites for therapeutic agents. The simplest form of DNA polypod could be built from three ODN strands. Comparing to DNA tetrahedron, polypod-shaped DNA has the same simple structural design yet better geometrical flexibility. The nanostructure could be functionalized with various motifs.

Polypodna itself could serve as an immunostimulatory agent. Y-shaped polypodna could induce great amounts of cytokines TNF-α and IL-6 than normal native double-stranded DNA (Yata et al., 2015). Y_L_-DNA (ligated Y-shaped DNA) also exhibits TLR9-mediated activation of DCs and macrophages, as revealed by promoted expression of the immune-relevant molecules (Yang et al., 2019). They further loaded CpG to amplify the effects and uptake efficiency. X-shaped DNA also serves as a favorable immune adjuvant that promotes the curative effect of anticancer drugs (Yang et al., 2019). Both X_S_-DNA (single unit of X-shaped DNA) and X_L_-DNA (ligated X-shaped DNA) induce the secretion of immune-relevant cytokines and costimulatory molecules in DCs, while the latter is more efficient. X_L_-DNA treatment of *in vitro* and *in vivo* results in the differentiation of naive CD4^+^ T cells into TH_1_ cells, and the combination of TLR9 and inflammasome greatly enhances the anticancer effect of Doxorubicin (Dox) in an animal model.

**Figure 9:**
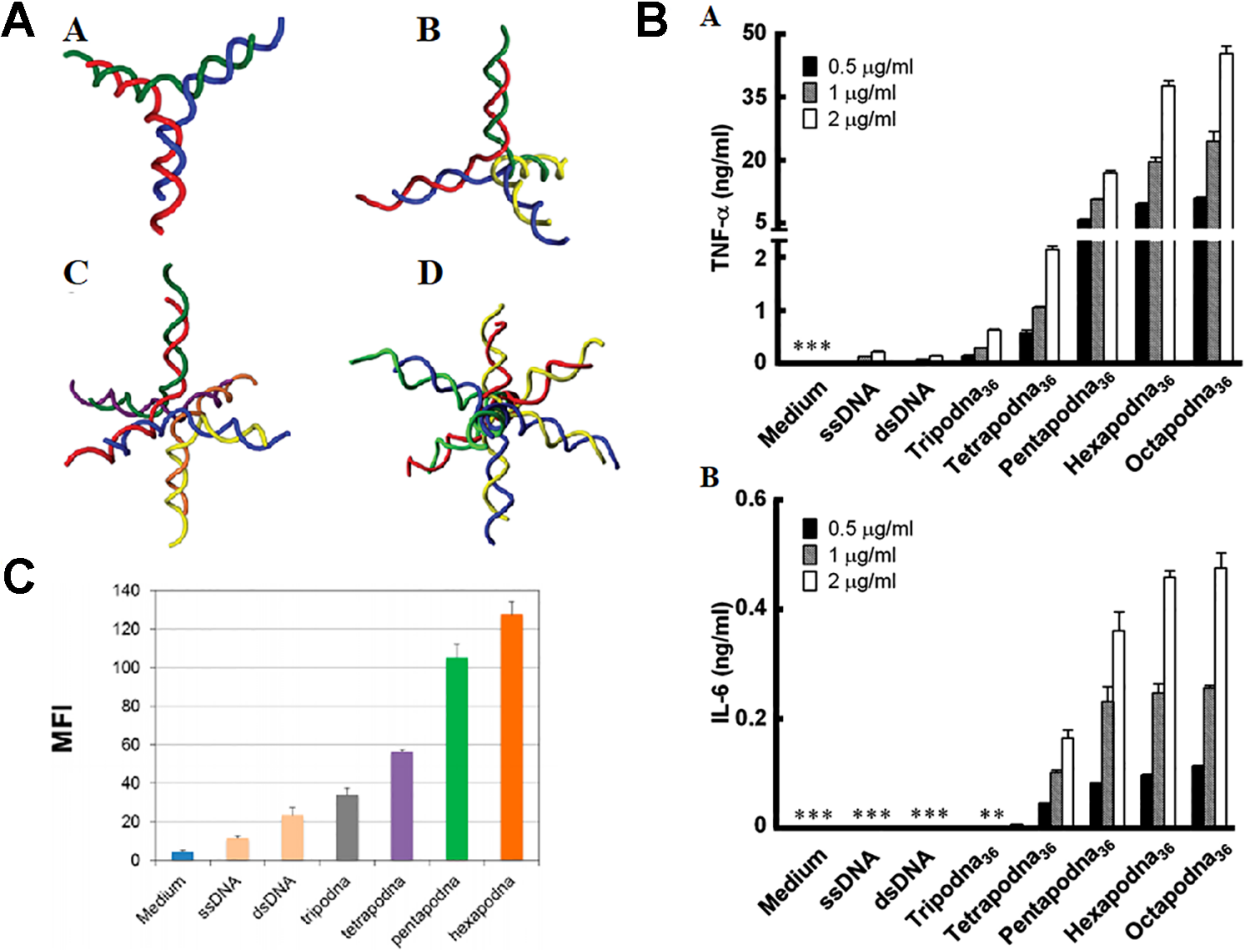
Therapeutic effect of various polypodna. **(A)** Schematic diagram of different structure polypodna. A tripodna; B tetrapodna; C hexapodna; and D octapodna (adapted with permission from [Mohri et al., 2012)]. **(B)** Production of (A) TNF-ɑ and (B) IL-6 from RAW264.7 cells. [adapted with permission from (Mohri et al., 2012)]. **(C)** Cellular uptake amount of single- or double-stranded DNAs, the polypodnas, and a medium without DNA [reproduced with permission from (Sanada et al., 2016)]. TNF-ɑ; tumor necrosis factor-ɑ.

Early in 2008, DNA polypods were equipped with CpG elements (Nishikawa et al., 2008). From then on, a series of polypod DNA has been constructed to load CpG. It was found such a complicated structured DNA serves as a highly efficient delivery system of CpG to TLR-positive immune cells (Nishikawa et al., 2008). Besides trigonal Y-shaped shape, polypods with a more complex branching structure in which CpG is contained were constructed to test the immunostimulatory activity (Mohri et al., 2012). A tri-, tetra-, hexa- and octapod DNA were prepared, as shown in **Figure 9A**. Each polypod DNA could induce the production of TNF-α and IL-6 from macrophage-like cells more intensely than double-stranded CpG-contained non-branched DNA. Increasing the number of pods promote immune reaction but reduced the stability, while hexa- and octapod DNA induced the most extensive response, as demonstrated in **Figure 9B**. Except for RAW264.7, studies of CpG-contained polypodna in other cells are also implemented. In addition to CpG, Polypodna can also be used to immunosuppress the delivery of ODN. Hexapodna, which is incorporated into the immunosuppressive agent A151, inhibits immune cell viability more effectively than A151 and effectively inhibits CpG ODN-induced cytokine release (Mohri et al., 2012). Uno et al. examined the immunostimulatory reaction of polypod DNA in various APCs and *in vivo via* injection into mice (Uno et al., 2014). The cellular uptake and cytokine release are confirmed to be proportional to the pod number, as shown in **Figure 9C** (Uno et al., 2014). Furthermore, they revealed polypod DNA generated much more IFN-α in human peripheral blood mononuclear cells in comparison with ssDNA.

**Figure 10:**
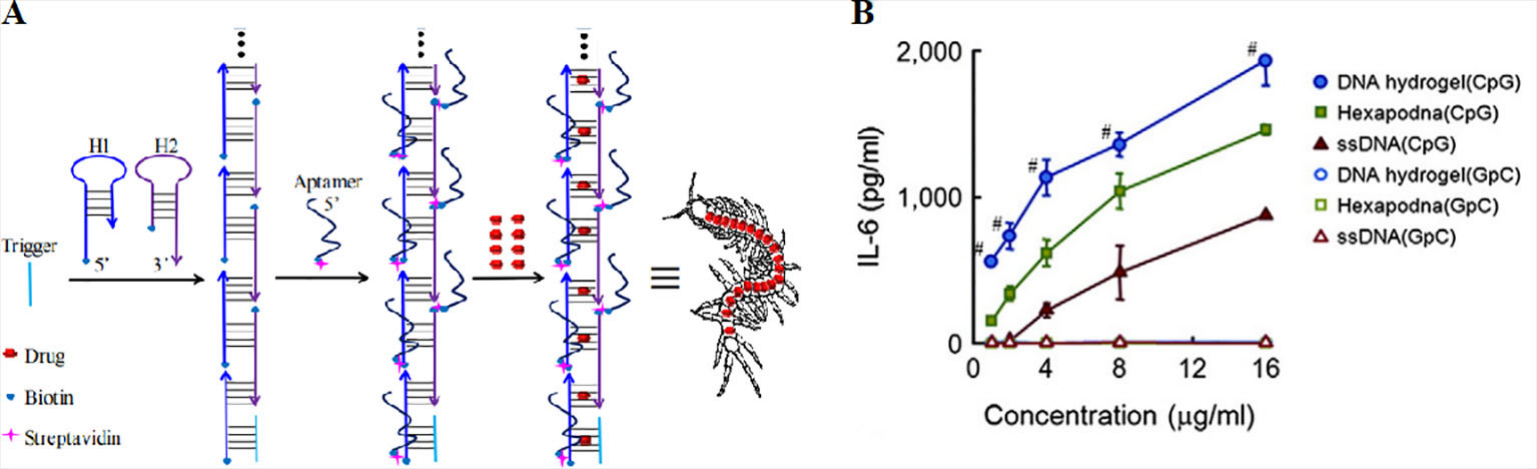
The structure of aptamer-based DNA nanocentipede and the function of polypodna hydrogel. **(A)** DNA Nanocentipede based on self-assembled aptamers can realize targeted delivery of the drug. [adapted with permission from (Li et al., 2016)]. **(B)** IL-6 production from DC2.4 cells by polypodna hydrogel (CpG) and other groups. [reproduced with permission from (Nishikawa et al., 2014)]. ^#^P < 0.05 compared with all others.

DNA nanocentipede was firstly introduced by Li et al. as a powerful delivery platform to deal with the challenges of targeted drug delivery (Li et al., 2017). The structure was similar to centipede and consist of “trunk” and “legs”, as shown in **Figure 10A**. The structure has a high capacity of the payload by fully loading the nanocentipede trunk with drug molecules. Nanocentipede trunk was prepared by assembling two short DNA monomers via HCR procedure. Meanwhile, nanocentipede legs were aptamers which serve as targeting moieties to target cells and they grasp target cells firmly to enable efficient uptake. The long trunk of DNA nanocentipede was loaded with Dox, while the legs are aptamers which selectively grasp target cells. The structure exhibited high loading capacity and promoted cytotoxicity only to target cancer cells. Moreover, the same group used DNA nanocentipedes as a vehicle to deliver the CpG motif (Li et al., 2017). The nanostructure was internalized by RAW264.7 cells.

## DNA Hydrogels

Hydrogels have been extensively utilized as biocompatible, high-capacity drug carrier 3D scaffolds in the field of biomedicine (Appel et al., 2014; Li and Mooney, 2016). DNA-based hydrogels have arrested researchers' attention due to the combined features brought by nucleic acids and hydrogels, including large sizes, good biocompatibility, flexibility and high-capacity (Costa et al., 2018). The hydrogel of polypod could be prepared by connecting multiple polypod DNA units, as firstly found by Um et al. (2006). DNA hydrogel of polypod has shown great potential in repressing tumor activity.

The hydrogel of DNA polypod integrated by CpG motifs was found to be more effective than the CpG-absent counterpart in the term of production of TNF-α from macrophages (Nishikawa et al., 2011). Dox was released slowly from the CpG-integrated polypod DNA hydrogel. The same group found the gel formation of CpG-incorporated polypod DNA essentially promoted the immunostimulatory activity (Nishikawa et al., 2014), as demonstrated in **Figure 10B**. The model antigen carried by hydrogel of hexapod DNA efficiently binds to mouse DCs and generates high antigen activity (Umeki et al., 2015). Intratumoral injections of the formulation prominently suppress tumor growth in mice.

A prominent problem with DNA hydrogels is their fast release rate, making it difficult to apply to controlled drug release. To overcome the problem of the rapid release of ordinary DNA hydrogels, size-controllable and stimuli-responsive DNA nanohydrogels haven been reported. Nanohydrogels, which refers to polymeric nanoparticles, have been regarded as a powerful drug carrier due to their high payload capacity, biocompatibility, flexibility, and mechanical stability (Li et al., 2015; Jiang et al., 2019). DNA nanohydrogels are randomly self-assembled from functional polymer blocks by manners including base-pairing hybridization, liquid crystallization (Li et al., 2015; Jiang et al., 2019). Bi et al. built DNA nanohydrogels self-assembled from components of DNA four-way junction (DNA-4WJ), which is prepared from liquid crystallization and dense packaging (Bi et al., 2015). The nanohydrogels are further integrated by aptamers, bioimaging components, and drug-loading sites for targeted therapy of cancer. Each DNA-4WJ unit provides ~30 loading sites for Dox. Its drug loading capacity is much larger than traditional DNA nanostructures. Imaging of confocal microscopy demonstrated selectively targeted transport of anticancer drug into human acute lymphoblastic leukemia cells rather than nontarget Ramos cells.

Sometimes, constructing DNA nanostructures through base-pairing of DNA sequences suffer from complicated design, tedious operation as well as low stability. DNA nanoflower is a kind of large-scale DNA hydrogels which does not rely on base-pairing interactions (Zhu et al., 2013; Lv et al., 2015). In comparison with the assembly of DNA hybridization, it is generated by RCR, along with liquid crystallization technique and dense packaging process (Lv et al., 2015). The nanostructure of the type has many advantages: simple design and preparation, large-scale tunable size, and resistance to enzymatic degradation (Lv et al., 2015; Park et al., 2017). Authors have built multifunctional DNA nanoflowers incorporated by the therapeutic drug, bioimaging agents, and genes (Hu et al., 2014; Mei et al., 2015; Yu et al., 2019).

**Figure 11:**
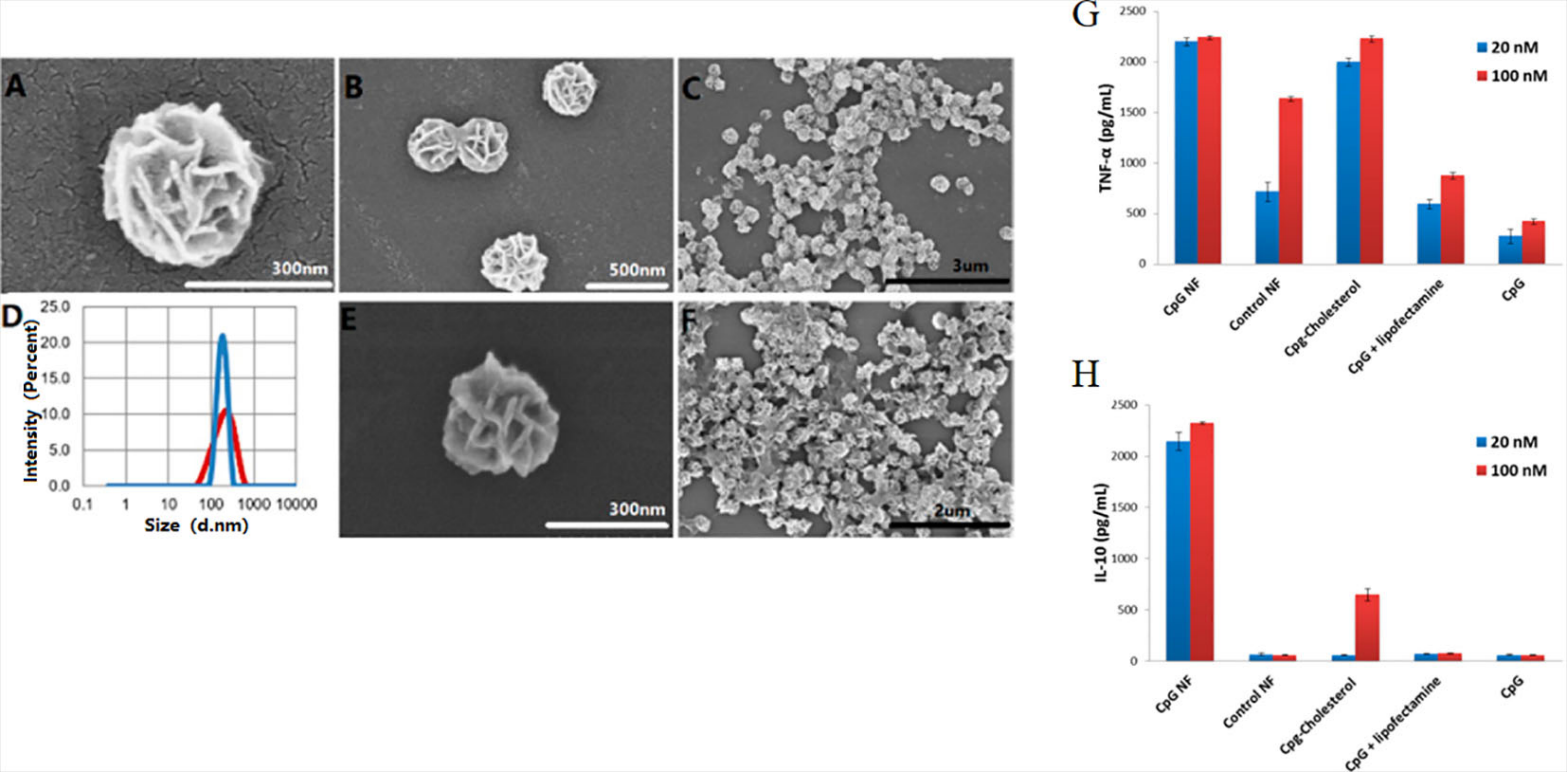
SEM observation and immunostimulatory function of DNA nanoflowers. **(A–C, E–F)** SEM images showing structures of nanoflowers at different scales. **(D)** Distribution of sizes of nanoflowers before (red) and after (blue) treatment with DNase I as measured by DLS. **(G, H)** Secretion of cytokine secreted by CpG-containing DNA nanoflowers and other control groups [reproduced with permission from (Zhang et al., 2015)].

Nanoflowers could be easily internalized by macrophages, which are important APCs. DNA nanoflowers could be easily internalized by macrophages due to their nanoscale size (**Figures 11A–F**). Authors integrated CpG into the multifunctional DNA nanoflowers to trigger immune reactions of co-cultured macrophage cells, inducing apoptosis and necrosis of cancer cells (Zhang F. et al., 2015). The results demonstrate DNA nanoflowers are an excellent nanocarrier for the intracellular delivery of CpG for immunotherapy strategies of cancer. These biocompatible nanoflowers are resistant to nuclease degradation. In a macrophage-like cell model, CpG nanoflowers secretes immunostimulatory cytokines, including tumor necrosis factor-alpha, by triggering these immune cells, interleukin-6, and interleukin-10 (**Figures 11G, H**).

## Conclusions and Future Outlook

The robust properties of DNA self-assembling allow for a programmable design of nanostructures with required sizes and functionality for the best performance of drug delivery. Abundant advantages make DNA nanostructures an ideal platform to deliver immune drugs, including CpG ODNs and other immunostimulatory agents, to target locations. Drug delivery systems based on various DNA nanostructure been proposed for the treatment of cancer and other diseases. DNA helices are densely packaged into 3D cages structures to prevent against DNA-degrading enzymes. DNA nanostructures of the type are compact, stable and are easily absorbed by cells. SNA relies on its unique 3D structure to carry a large amount of CpG, and can easily enter most of the immune cells to implement immunotherapy for a variety of diseases. DNA-based nanoparticles are ideal for developing DNA vaccines due to that the shape of these nanoparticles is similar to that of virus particles. DNA nanoparticles can also integrate other functional ingredients for more effective immunotherapy. Moreover, polypod-like DNA nanostructures take advantage of the structural features to provide large docking sites for immune drugs. Ordinary DNA hydrogel is characteristic of large sizes and high payload. Authors handled the problem of rapid release to implement efficient drug delivery. Other kinds of DNA hydrogels, including DNA nanohydrogels and DNA nanoflowers, also witnessed their characteristics of stability, payload, and cellular uptake. It should be noted that the application of different DNA nanostructures are different in immunotherapy, as shown in **Table 1**.

**Table 1:** Application of different DNA nanostructures in immunotherapy.

Type of DNA Nanostructures	Specific type	Applications
Wireframe DNA cage	DNA polyhedrons DNA nanotubes 用	DNA vaccines Activation of APCs Eliciting immunogenic responses *in vivo* Long retention time
Spherical nucleic acids	Traditional SNAs Liposomal SNAs Cholesterol-tail LSNA Dual TLR targeting LSNA	Activation of APCs Immunomodulation Reduce fibrosis of nonalcoholic steatohepatitis Repress tumor growth Improve macrophage bactericidal activity
Hybrid DNA-based nanoparticles	Metal phosphate nanoparticles DNA-lipid micelle nanoparticles DNA-RNA nanocapsules	Activation of APCs Transfection *in vitro*/*in vivo* DNA vaccine Immune stimulation of DCs *in vivo* Repress tumor growth
Polypod-like DNA	Polypodna DNA nanocentipede	Activation of APCs Immunosuppression Promote the curative effect of anticancer drugs Differentiation of naive CD4^+^ T cells into TH_1_ cells
DNA hydrogels	Polypod hydrogel DNA nanohydrogels DNA nanoflowers	Activation of APCs Slow down release of Dox Suppress tumor growth *in vivo* Induce apoptosis and necrosis of cancer cells

Despite significant advances in building DNA nanostructure-based drug delivery systems, there are still challenges that hinder their further applications for immunotherapy. Production costs and purification procedures remain an obstacle. Moreover, the native immune system is quite resistant to foreign DNA nanodevices and may quickly eliminate them. Therefore, efforts should be made to develop more biocompatible DNA nanostructures. DNA nanostructures with high biocompatibility are ideal for extended retention in applications *in vivo*. The use of biomimetic DNA nanostructures that mimic natural materials can help them escape the immune system (Perrault and Shih, 2014; Hu et al., 2015). DNA nanostructures composed of abundant foreign nucleic acids may pose potential safety concerns. Importantly, biosafety must be confirmed prior to applications *in vivo*. Therefore, different levels of toxicity comprehensive experimental evaluation from cell to animal experiments are needed. Therefore, systematic toxicity assessments must be performed, including long-term toxicity, pharmacokinetics, and biodegradability.

It should be noted that there are a series of biological barriers in the transmission of DNA nanostructures, including biological mucosa, lysosomal phagocytosis, and cell membrane internalization. These barrier structures can severely hamper the use of DNA nanodevices. Therefore, future research should pay more attention to designing nanostrucures that can continuously overcome various obstacles. Investigators sometimes choose to develop flexible, intelligent DNA nanodevices that overcome obstacles (Akita and Harashima, 2008; Blanco et al., 2015).

It must be noted that immunotherapy based on DNA nanostructures is still in early stages and more efforts are needed to advance the field. At present, many excellent DNA nanostructures for immunotherapy have been established *in vitro*, but similar devices for drug delivery *in vivo* are still lacking. Great efforts are required to develop highly reliable and stable DNA nanostructures for application *in vivo*. In order to determine long-term biocompatibility at different levels, including cells, tissues, organs and animals, there is still a considerable amount of work to be done. On the other hand, the characterization of DNA nanostructures in different experiments may suggest conflicting results. Therefore, it is important to establish standardized and reliable methods for evaluating efficacy. In addition, in order to implement more effective immunotherapy, there is an urgent need to develop standardized DNA nanostructures suitable for clinical treatment of specific diseases.

## Author Contributions

QC implemented the analysis and wrote the manuscript. ZY contributed to the discussion of the paper. KX and CW provided some analysis of the results. HL proposed the idea of the manuscript.

## Funding

This study was supported by grants from the National Natural Science Foundation of China (11602181), the Fundamental Research Funds for the Central Universities (WUT: 2018IB005), the Open Project of the State Key Laboratory of Trauma, Burn and Combined Injury, Army Medical University (NO. SKLKF201606), the Visiting Scholar Foundation of Key Laboratory of Biorheological Science and Technology (Chongqing University), Ministry of Education (Grant Number: CQKLBST-2018-006, CQKLBST-2018-009).

## Conflict of Interest

The authors declare that the research was conducted in the absence of any commercial or financial relationships that could be construed as a potential conflict of interest.

## Abbreviations

aPD1, anti-PD-1 antibodies; APCs, antigen-presenting cells; CaPi, calcium phosphate; DCs, dendritic cells; DNA-4WJ, DNA four-way junction; DNCs, DNA nano-cocoons; Dox, Doxorubicin; HCR, hybridization chain reaction; iDR-NC, intertwining DNA-RNA nanocapsules; IFN-α/γ, interferon-α/γ; IL-6, interleukin-6; IL-12, interleukin-12; LPD, lipid-protamine-DNA; LSNAs, Liposomal spherical nucleic acids; NASH, nonalcoholic steatohepatitis; ODNs, Oligodeoxynucleotides; pEGFP, plasmid DNA expressing an enhanced green fluorescent protein; PLGA, poly(lactic-co-glycolic acid); Polypodna, polypod DNA; PR-NC, photoresponsive nanocapsule; PTO, phosphorothioate; RCR, rolling-circle replication; RCT, rolling circle transcription; shRNA, short hairpin RNA; siRNA, small interference RNA; SNA, spherical nucleic acids; ssDNA, single-stranded DNA; STV, streptavidin; TLR3/4/7/8/9, Toll-like receptor 3/4/7/8/9; TNF-α, tumor necrosis factor-α.
